# Virulence factors and antibiotic resistance properties of the *Staphylococcus epidermidis* strains isolated from hospital infections in Ahvaz, Iran

**DOI:** 10.1186/s41182-019-0180-7

**Published:** 2019-12-05

**Authors:** Roya Chabi, Hassan Momtaz

**Affiliations:** Department of Microbiology, Shahrekord Branch, Islamic Azad University, PO. Box: 166, Shahrekord, Iran

**Keywords:** *Staphylococcus epidermidis*, Antibiotic resistance, Antibiotic resistance genes, Hospital infections, Iran

## Abstract

**Background:**

Resistant *Staphylococcus epidermidis* strains are considered to be one of the major causes of human clinical infections in hospitals. The present investigation was done to study the pattern of antibiotic resistance and the prevalence of virulence and antibiotic resistance genes amongst the *S. epidermidis* strains isolated from human hospital infections.

**Methods:**

One hundred hospital infectious samples were collected and *S. epidermidis* strains were identified using culture and biochemical tests. Isolated strains were subjected to disk diffusion and PCR.

**Results:**

Forty-six out of 100 hospital infectious samples (46%) were positive for *S. epidermidis*. *S. epidermidis* strains harbored the highest prevalence of resistance against penicillin (95.65%), tetracycline (91.30%), erythromycin (82.60%), cefazolin (78.26%), and trimethoprim-sulfamethoxazole (73.91%). All *S. epidermidis* strains had resistance against at least three different types of antibiotics, while the prevalence of resistance against more than seven types of antibiotics was 17.39%. *AacA-D* (69.56%), *tetK* (56.52%), *mecA* (45.65%), *msrA* (39.13%), and *tetM* (39.13%) were most commonly detected antibiotic resistance genes. The prevalence of *vatC* (4.34%), *ermA* (8.69%), *vatA* (8.69%), *vatB* (13.04%), *ermC* (13.04%), and *linA* (10.86%) were lower than other detected antibiotic resistance genes. *ClfA* (32.60%), *agrIII* (17.39%), and *etB* (13.04%) were the most commonly detected virulence factors.

**Conclusions:**

The presence of virulent and multi-drug resistance *S. epidermidis* strains showed an important public health issue in hospitals.

## Background

Hospital infections are considered as a major issue all around the world. Bacteria have the highest impact on the occurrence of hospital infections [1–7]. *Staphylococcus* spp. are commensal bacteria of human skin and have been isolated from diverse clinical sources such as urinary tract infections (UTIs), respiratory tract infections (RTIs), wound infections (WIs), soft tissue infections, blood infections, and endocarditis [3, 8–10]. It has been suggested that *Staphylococcus epidermidis* (*S. epidermidis*) is one of the most important species of this group. It is a Gram-positive, non-spore forming, nonmotile, facultative anaerobic, and catalase-positive and coagulase-negative bacterium responsible for different types of hospital and nosocomial infections. Indwelling medical devices are considered a major vector of *S. epidermidis* in hospitalized patients [11, 12]. *S. epidermidis* results in approximately 13% of prosthetic valve endocarditis infections, with a high rate of intracardiac abscess formation (38%) and mortality (24%) [13].

*S. epidermidis* strains usually resist against several types of antibiotic classes such as tetracyclines, aminoglycosides, cephalosporins, fluoroquinolones, penicillins, and macrolides [14–17]. Nowadays, resistant *S. epidermidis* has become a serious problem in hospitals [14–16]. Resistant staphylococcal strains are responsible for about 100,000 cases of infections with around 10% mortality rate each year in the USA [16]. The presence of certain antibiotic resistance genes is responsible for the occurrence of antibiotic resistance [14–17]. *MecA*, *aacA-D*, *tetK* and *tetM*, *ermA* and *ermC*, *msrA* and *msrB*, *linA* and *vatA*, and *vatB* and *vatC* antibiotic resistance genes are responsible for the occurrence of resistance against methicillin, aminoglycosides, tetracyclines, macrolide-lincosamide-streptogramin B, macrolides, lincosamides, and streptogramin A groups of antibiotics, respectively [3, 14–17] (Table 1).

**Table 1:** Antibiotic resistance pattern of the *S. epidermidis* strains isolated from different types of hospital infectious samples

Samples (no. positive samples for *S. epidermidis*)	Antibiotic resistance pattern (%)										
	P10*	Cef	Cip5	Clin	Az	Eryt	Mup	Rif	Tet30	Tri-Sul	N/F300
UTIs (10)	9 (90)	7 (70)	5 (50)	5 (50)	5 (50)	8 (80)	2 (20)	5 (50)	8 (80)	6 (60)	2 (20)
WIs (20)	19 (95)	16 (80)	15 (75)	13 (65)	12 (60)	16 (80)	11 (55)	12 (60)	19 (95)	15 (75)	8 (40)
RTIs (16)	16 (100)	13 (81.25)	12 (75)	12 (75)	11 (68.75)	14 (87.50)	10 (62.50)	11 (68.75)	15 (93.75)	13 (81.25)	6 (37.50)
Total (46)	44 (95.65)	36 (78.26)	32 (69.56)	30 (65.21)	28 (60.86)	38 (82.60)	23 (50)	28 (60.86)	42 (91.30)	34 (73.91)	16 (34.78)

*P10 penicillin (10 μg/disk), *Cef* cefazolin (30 μg/disk), *Cip5* ciprofloxacin (5 μg/disk), *Clin* clindamycin (2 μg/disk), *Az* azithromycin (15 μg/disk), *Eryt* erythromycin (15 μg/disk), *Mup* mupirocin (30 μg/disk), *Rif* rifampin (5 μg/disk), *Tet30* tetracycline (30 μg/disk), *Tri-Sul* trimethoprim-sulfamethoxazole (25 μg/disk), and *N/F300* nitrofurantoin (300 μg/disk) antibiotic agents

Some potential virulence factors including toxic shock syndrome toxin-1 (TSST-1 encoded by *tst*), exfoliative toxins A and B (*eta* and *etb*), clumping factor (*clfA*), and types I, II, and III of the accessory gene regulator (*agr*) are responsible for virulence characters of the *S. epidermidis* strains isolated from human clinical infections [10]. The *X-region* gene of *Staphylococcus* strains has a high degree of importance in the occurrence of diseases, and it may have a variation rate (or clock speed) that provides suitable discrimination for outbreak investigation [10]. The *IgG-binding region* is responsible for causing host specificity and various immunological responses against *Staphylococcus* strains. The *X-region* and *IgG-binding region* have been detected in various types of staphylococcal infections [10].

Scarce researches have been conducted on epidemiological and molecular aspects of the *S. epidermidis* strains in hospital infections in Iran. Thus, the current research was done to study the prevalence rate, distribution of virulence factors, and antimicrobial resistance properties of *S. epidermidis* strains isolated from various types of human clinical infections collected from Ahvaz city, Iran.

## Results

The present investigation was done to assess the antibiotic resistance properties and distribution of virulence genes amongst the *S. epidermidis* strains isolated from different types of hospital infectious samples. Forty-six out of 100 hospital infectious samples (46%) were positive for *S. epidermidis*.

Table 2 represents the antibiotic resistance pattern of the *S. epidermidis* strains isolated from hospital infectious samples. *S. epidermidis* strains harbored the highest prevalence of resistance against penicillin (95.65%), tetracycline (91.30%), erythromycin (82.60%), cefazolin (78.26%), and trimethoprim-sulfamethoxazole (73.91%) antibiotic agents. Reversely, *S. epidermidis* strains harbored the lowest prevalence of resistance against nitrofurantoin (34.78%) and mupirocin (50%) antibiotic agents. The prevalence of resistance against ciprofloxacin, clindamycin, azithromycin, and rifampin antibiotic agents were 69.56%, 65.21%, 60.86%, and 60.86%, respectively.

**Table 2:** Distribution of antibiotic resistance genes amongst the *S. epidermidis* strains isolated from different types of hospital infectious samples

Samples (no. positive samples for *S. epidermidis*)	Antibiotic resistance genes (%)											
	*mecA*	*msrA*	*msrB*	*AacA-D*	*tetK*	*tetM*	*vatA*	*vatB*	*vatC*	*ermA*	*ermC*	*linA*
UTIs (10)	2 (20)	2 (20)	1 (10)	5 (50)	4 (40)	2 (20)	1 (10)	1 (10)	–	1 (10)	1 (10)	1 (10)
WIs (20)	10 (50)	9 (45)	7 (35)	16 (80)	12 (60)	8 (40)	2 (10)	3 (15)	1 (5)	2 (10)	3 (15)	3 (15)
RTIs (16)	9 (56.25)	7 (43.75)	4 (25)	11 (68.75)	10 (62.50)	8 (50)	1 (6.25)	2 (12.50)	1 (6.25)	1 (6.25)	2 (12.50)	1 (6.25)
Total (46)	21 (45.65)	18 (39.13)	12 (26.08)	32 (69.56)	26 (56.52)	18 (39.13)	4 (8.69)	6 (13.04)	2 (4.34)	4 (8.69)	6 (13.04)	5 (10.86)

**Fig. 1:**
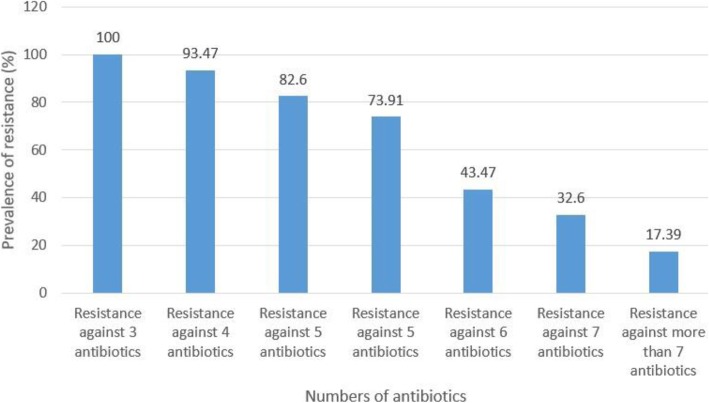
Prevalence of multidrug-resistant *S. epidermidis* strains in hospital infectious samples

Figure 1 represents the prevalence of multi-drug resistant *S. epidermidis* strains isolated from hospital infectious samples. Multidrug-resistant *S. epidermidis* strains were determined as those who had at least simultaneous resistance against three or more than three types of antibiotics. All *S. epidermidis* strains had resistance against at least three different types of antibiotics, while the prevalence of resistance against more than seven types of antibiotics was 17.39%.

Table 3 represents the distribution of antibiotic resistance genes amongst the *S. epidermidis* strains isolated from hospital infectious samples. We found that *aacA-D* (69.56%), *tetK* (56.52%), *mecA* (45.65%), *msrA* (39.13%), and *tetM* (39.13%) were most commonly detected antibiotic resistance genes amongst the *S. epidermidis* strains. The prevalence of *vatC* (4.34%), *ermA* (8.69%), *vatA* (8.69%), *vatB* (13.04%), *ermC* (13.04%), and *linA* (10.86%) were lower than other detected antibiotic resistance genes. The prevalence of *msrA* and *msrB* antibiotic resistance genes were 36.13% and 26.08%, respectively.

**Table 3:** Distribution of virulence factors amongst the *S. epidermidis* strains isolated from different types of hospital infectious samples

Samples (no. positive samples for *S. epidermidis*)	Virulence factors (%)									
	*coa*	*clfA*	*X-region*	*IgG-binding region*	*tsst-1*	*etA*	*etB*	*agrI*	*agrII*	*agrIII*
UTIs (10)	–	2 (20)	–	–	–	–	1 (10)	1 (10)	1 (10)	2 (20)
WIs (20)	–	8 (40)	–	–	1 (5)	2 (10)	3 (15)	2 (10)	2 (10)	4 (20)
RTIs (16)	–	5 (31.25)	–	–	1 (6.25)	1 (6.25)	2 (12.50)	1 (6.25)	2 (12.50)	2 (12.50)
Total (46)	–	15 (32.60)	–	–	2 (4.34)	3 (6.52)	6 (13.04)	4 (8.69)	5 (10.86)	8 (17.39)

*tetK* (56.52%), *mecA* (45.65%), *msrA* (39.13%), and *tetM* (39.13%) were most commonly detected antibiotic resistance genes. The prevalence of *vatC* (4.34%), *ermA* (8.69%), *vatA* (8.69%), *vatB* (13.04%), *ermC* (13.04%), and *linA* (10.86%) were lower.

Table 4 represents the distribution of virulence factors amongst the *S. epidermidis* strains isolated from hospital infectious samples. We found that *clfA* (32.60%), *agrIII* (17.39%), and *etB* (13.04%) were the most commonly detected virulence factors amongst the *S. epidermidis* strains isolated from hospital infectious samples. Virulence factors for *coa*, *X-region*, and *IgG-binding region* were negative.

**Table 4:** Target genes, oligonucleotide primers, and PCR conditions used for the detection of virulence factors and antibiotic resistance genes in the *S. epidermidis* strains isolated from various types of hospital infectious samples

Target gene	Primer sequence (5′-3′)	PCR product (bp)	PCR programs	PCR volume (50 μL)
*vatA*	F: TGGTCCCGGAACAACATTTAT R: TCCACCGACAATAGAATAGGG	268	1 cycle: 94 °C, 5 min 30 cycles: 94 °C, 60 s 60 °C, 60 s 72 °C, 90 s 1 cycle: 72 °C, 7 min	5 μL PCR buffer 10X 1.5 mM Mgcl_2_ 200 μM dNTP (Fermentas) 0.5 μM of each primers F & R 1.25 U Taq DNA polymerase (Fermentas) 2.5 μL DNA template
*vatB*	F: GCTGCGAATTCAGTTGTTACA R: CTGACCAATCCCACCATTTTA	136		
*vatC*	F: AAGGCCCCAATCCAGAAGAA R: TCAACGTTCTTTGTCACAACC	467		
*mecA*	F: AAAATCGATGGTAAAGGTTGGC R: AGTTCTGCAGTACCGGATTTGC	532		
*tetK*	F: GTAGCGACAATAGGTAATAGT R: GTAGTGACAATAAACCTCCTA	360		
*tetM*	F: AGTGGAGCGATTACAGAA R: CATATGTCCTGGCGTGTCTA	158	1 cycle: 94 °C, 5 min 30 cycles: 94 °C, 60 s 60 °C, 60 s 72 °C, 90 s 1 cycle: 72 °C, 7	5 μL PCR buffer 10X 2 mM Mgcl_2_ 200 μM dNTP (Fermentas) 0.5 μM of each primers F & R 1.5 U Taq DNA polymerase (Fermentas) 5 μL DNA template
*msrA*	F: GGCACAATAAGAGTGTTTAAAGG R: C AAGTTATATCATGAATAGATTGTCCTGTT	940		
*msrB*	F: TATGATATCCATAATAATTATCCAATC R: AAGTTATATCATGAATAGATTGTCCTGTT	595		
*aacA-D*	F: TAATCCAAGAGCAATAAGGGC R: GCCACACTATCATAACCACTA	227		
*ermA*	F: AAGCGGTAAACCCCTCTGA R: TTCGCAAATCCCTTCTCAAC	190		
*ermC*	F: AATCGTCAATTCCTGCATGT R: AATCGTCAATTCCTGCATGT	229		
*linA*	F: GGTGGCTGGGGGGTAGATGTATTAACTGG R: GCTTCTTTTGAAATACATGGTATTTTTCGA	323		
*tsst-1*	F: ATGGCAGCATCAGCTTGATA R: TTTCCAATAACCACCCGTTT	350	1 cycle: 94 °C, 6 min 30 cycles: 94 °C, 2 min 55 °C, 2 min 72 °C, 1 min 1 cycle: 72 °C, 8 min	5 μL PCR buffer 10X 2 mM Mgcl_2_ 200 μM dNTP (Fermentas) 0.5 μM of each primers F & R 1.5 U Taq DNA polymerase (Fermentas) 5 μL DNA template
*etA*	F: CTAGTGCATTTGTTATTCAA R: TGCATTGACACCATAGTACT	119		
*etB*	F: ACGGCTATATACATTCAATT R: TCCATCGATAATATACCTAA	200		
*agrI*	F: ATGCACATGGTGCACATGC R: GTCACAAGTACTATAAGCTGCGAT	441	1 cycle: 94 °C, 6 min 26 cycle: 94 °C, 30 s 55 °C, 30 s 72 °C, 1 min 1 cycle: 72 °C, 8 min	5 μL PCR buffer 10X 2 mM Mgcl_2_ 200 μM dNTP (Fermentas) 0.5 μM of each primers F & R 1.5 U Taq DNA polymerase (Fermentas) 5 μL DNA template
*agrII*	F: ATGCACATGGTGCACATGC R: TATTACTAATTGAAAAGTGGCCATAGC	575		
*agrIII*	F: ATGCACATGGTGCACATGC R: GTAATGTAATAGCTTGTATAATAATACCCAG	323		
*coa*	F: CGAGACCAAGATTCAACAAG R: AAAGAAAACCACTCACATCA	970	1 cycle: 95 °C, 2 min 30 cycles: 95 °C, 30 s 58 °C, 2 min 72 °C, 4 min 1 cycle: 72 °C, 7 min	5 μL PCR buffer 10X 2 mM Mgcl_2_ 200 μM dNTP (Fermentas) 0.5 μM of each primers F & R 1.5 U Taq DNA polymerase (Fermentas) 5 μL DNA template
*clfA*	F: GGCTTCAGTGCTTGTAGG R: TTTTCAGGGTCAATATAAGC	980	1 cycle: 94 °C, 4 min 35 cycles: 94 °C, 1 min 57 °C, 1 min 72 °C, 1 min 1 cycle: 72 °C, 5 min	5 μL PCR buffer 10X 2 mM Mgcl_2_ 200 μM dNTP (Fermentas) 0.5 μM of each primers F & R 1.5 U Taq DNA polymerase (Fermentas) 5 μL DNA template
*X-region*	F: CAAGCACCAAAAGAGGAA R: CACCAGGTTTAACGACAT	320	1 cycle: 95 °C, 4 min 25 cycles: 95 °C, 1 min 60 °C, 1 min 72 °C, 1 min 1 cycle: 72 °C, 3 min	5 μL PCR buffer 10X 2 mM Mgcl_2_ 200 μM dNTP (Fermentas) 0.5 μM of each primers F & R 1.5 U Taq DNA polymerase (Fermentas) 5 μL DNA template
*IgG-binding region*	F: CACCTGCTGCAAATGCTGCG R: GGCTTGTTGTTGTCTTCCTC	920	1 cycle: 94 °C, 2 min 30 cycles: 94 °C, 1 min 58 °C, 1 min 72 °C, 1 min 1 cycle: 72 °C, 5 min	5 μL PCR buffer 10X 2 mM Mgcl_2_ 200 μM dNTP (Fermentas) 0.5 μM of each primers F & R 1.5 U Taq DNA polymerase (Fermentas) 5 μL DNA template

## Discussion

*S. epidermidis* is a common commensal bacterium of the human skin and mucosa. While *S. epidermidis* has long been considered nonpathogenic, it is now recognized as a relevant opportunistic pathogen [11, 12, 14]. Most *S. epidermidis*-related not only are associated with intravascular devices (prosthetic heart valves, shunts, etc.) but also commonly occur in prosthetic joints, catheters, and large wounds. However, recently published data revealed the high prevalence of *S. epidermidis* in the cases of human clinical infections [11, 12, 14]. Additionally, nosocomial *S. epidermidis* isolates were characterized by their pronounced resistance against many commonly used antibiotics [11, 12, 14].

The present study was done to assess the antibiotic resistance pattern and distribution of antibiotic resistance and virulence genes amongst the *S. epidermidis* strains isolated from different types of human clinical infections. Findings showed that 46% of human clinical infection samples were positive for *S. epidermidis* strains. Relatively high prevalence of *S. epidermidis* in the human clinical infections of the present study is may be due to the ubiquitous presence of the bacterium in the hospital environment, its ability to biofilm formation, and finally failure to observe sanitary and disinfection principles in hospitals in Ahwaz city, Iran. Because of the ubiquitous prevalence of *S. epidermidis* as a commensal bacterium, it is often difficult for a clinician to decide whether an isolate represents the causative agent of an infection or an unspecific culture contamination.

*S. epidermidis* strains harbored the highest prevalence of resistance against penicillin, tetracycline, erythromycin, cefazolin, and trimethoprim-sulfamethoxazole antibiotic agents. All *S. epidermidis* strains had resistance against at least three different types of antibiotics. Unauthorized and illegal prescription of antibiotics is the main reason for the high prevalence of antibiotic resistance. Mohaghegh et al. [18] reported that the prevalence of antibiotic resistance of the *S. aureus* strains against ampicillin, amoxicillin-clavulanic acid, cefepime, ceftazidime, nalidixic acid, and penicillin were 85%, 80%, 100%, 98.30%, 90.90%, and 90%, respectively. Moreover, the prevalence of resistance against tetracycline (51%), erythromycin (41.20%), oxacillin (97.70%), cephazolin (58.30), and norfloxacin (51.90%) was entirely high [18]. Eladli et al. [19] reported that the prevalence of antibiotic resistance of the *S. epidermidis* strains against amoxicillin-clavulanic acid, ciprofloxacin, clindamycin, erythromycin, gentamicin, levofloxacin, mupirocin, oxacillin, rifampin, tetracycline, and trimethoprim-sulfamethoxazole antibiotics was 100%, 100%, 37%, 0%, 33%, 16%, 0%, 80%, 0%, 80%, and 0%, respectively. Ma et al. [20] reported that the prevalence of antibiotic resistance of the coagulase-negative staphylococci strains against penicillin, oxacillin, erythromycin, tetracycline, clindamycin, ciprofloxacin, trimethoprim-sulfamethoxazole, chloramphenicol, ceftizoxime, gentamicin, rifampin, teicoplanin, and vancomycin antibiotics were 94.20%, 79.10%, 89.50%, 59.50%, 53.70%, 52.80%, 58.50%, 39.10%, 26.70%, 29.50%, 18.40%, 2.30, and 0%, respectively. High prevalence of multidrug-resistant *S. epidermidis* was also reported in their investigation. Similar patterns of antibiotic resistance of the *S. epidermidis* strains were reported from Mexico [21], Spain [22], Iran [23], USA [24], Belgium [25], and Ireland [26].

*S. epidermidis* strains isolated from the clinical infection samples harbored the high distribution of antibiotic resistance genes, especially *aacA-D*, *tetK*, *mecA*, and *tetM*. *S. epidermidis* strains had a considerable prevalence of resistance against clindamycin (65.21%). One of the most imperative mechanisms involving resistance against clindamycin is modulated by methylase enzyme which is often encoded by *ermA* and *ermC* genes [27]. The prevalence of *ermA* and *ermC* antibiotic resistance genes amongst the *S. epidermidis* strains of our research were 8.69% and 10.86%, respectively. The majority of isolates carried two tetracyclines, two erythromycins, one macrolide, and several streptogramin resistance determinants revealed a great diffusion of these types of resistance. The literature-based studies did not indicate any report on the prevalence of *vatA*, *vatB*, *vatC*, *msrA*, *ermA*, *ermC*, *linA*, *aacA-D*, *tetK*, and *tetM* antibiotic resistance genes amongst the *S. epidermidis* strains isolated from hospital infectious samples. Eksi [28] revealed the higher prevalence of *ermA* than *ermc* antibiotic resistance genes amongst the clindamycin, erythromycin, and telithromycin-resistant and also higher prevalence of *tetM* than *tetK* antibiotic resistance genes amongst the tetracycline-resistant MRSA strains. Duran et al. [17] reported that the distribution of *mecA*, *femA*, *ermA*, *ermB*, *ermC*, *tetK*, *tetM*, *msrA*, and *blaZ* antibiotic resistance genes amongst the coagulase negative staphylococci strains isolated from human clinical infections were 29.60%, 7.50%, 33.10%, 5.80%, 21.60%, 13.70%, 28.80%, 9.40%, and 93.50%, respectively. Adwan et al. [29] reported that the prevalence of *ermA*, *ermC*, *tetK*, *tetM*, *aacA-aphD*, *vatA*, *vatB*, and *vatC* genes amongst the staphylococci strains isolated from human infections were 30.90%, 74.50%, 76.40%, 16.40%, 74.50%, 1.80%, 0%, and 5.50%, respectively. High prevalence of *tetK* and *tetM* antibiotic resistance genes in the *S. epidermidis* isolates can be clarified by their usual genetic locations. The presence of *tetK* gene on small multicopy plasmids and *tetM* on conjugative transposons contribute to the spread of these determinants [30]. Some of the *S. epidermidis* strains harbored *ermC* gene. This gene is often located on small multicopy plasmids which are present in many different staphylococcal species [30]. The *ermA* gene is usually carried by transposons which could explain its high prevalence amongst the *S. epidermidis* strains. Resistance to aminoglycosides which are encoded by the *aacA-D* gene (69.59%) is more prevalent. It is because this gene is usually more diffused in staphylococci of human origin [30].

Amongst all virulence markers found in the *S. epidermidis* strains, genes encoding clumping factor (*clfA*), *IgG-binding region*, toxic shock syndrome toxin (*tst*), exfoliative toxins (*eta* and *etb*), accessory gene regulator (*agr*), and *X-region* were recognized as the most important markers in occurrence of infectious diseases caused by *S. epidermidis* [10]. Eftekhar et al. [31] reported that the frequency of the spa, *fnbB*, *fnbA*, *clfB*, *clfA*, *can*, *bbp*, *ebp*, *etb*, *eta*, *pvl*, and *tst* virulence genes amongst the *S. aureus* strains isolated from hospitalized patients was 100%, 75.70%, 74.30%, 78.60%, 71.40%, 24.30%, 0%, 58.60%, 2.90%, 7.10%, 21.40%, and 51.40%, respectively. Additionally, amongst all the examined genes, *clfB* (78.60%) and *etb* (2.90%) had the highest and lowest prevalence, respectively. The prevalence of *tsst-1* gene amongst the *S. epidermidis* strains of our research was low (4.34%). Similar findings have also been reported from Iran (11.60%) [32], Sweden (22.00%) [33], Malaysia (0.50%) [34], and Colombia (10.00%) [35]. *Tsst-1* gene is a pyrogenic toxin that encodes a 21.9 KDa extracellular toxin causing toxic shock syndrome (TSS). It is known as a severe acute disease distinguished by symptoms such as fever, rash, hypotension, and dysfunction of multiorgan systems. In addition, TSS secretion into the human blood may raise the rate of neonatal TSS-like exanthematous disease, Kawasaki syndrome, and sudden infant death syndrome [32]. Regarding the other detected genes, the *eta* gene was presented in 6.52% of strains. The prevalence of *etb* gene was 13.04%. The incidence rate of the *eta* and *etb* in the present study was higher than that reported in other investigations conducted on Iran (0.68%) [32], Colombia (3.00%) [35], and Malaysia (0%) [34]. A higher prevalence of *eta* gene was reported in studies conducted in Czech (10.00%) [36] and Turkey (19.20%) [37]. It was detected that the prevalence of the *etb* gene differs amongst numerous investigations, ranging from 0% in Colombia [35] and Malaysia [34] to 9.20% in Turkey [37]. Ghasemian et al. [38] reported the high prevalence of the *clfA* and *clfB* genes (100%). The incidence of the *clfA* gene in the bacterial strains of our research was relatively high (32.60%). A higher prevalence of this gene was reported from Brazil [39] and China [40]. Another important detected gene amongst the *S. epidermidis* strains was *agr*. The prevalence of *agrI*, *agrII*, and *agrIII* virulence genes amongst the *S. epidermidis* strains were 8.69%, 10.86%, and 17.39%, respectively. *Agr* virulence gene was also predominant amongst the *S. epidermidis* strains isolated from clinical samples recovered from China [40], USA [41], and Iran [42]. The accessory gene regulator (*agr*) locus influences the expression of many virulence genes in the *S. epidermidis*. Four allelic groups of *agr*, which generally inhibit the regulatory activity of each other, have been identified within the species. Interference in virulence gene expression caused by different *agr* groups has been suggested to be a mechanism for isolating bacterial populations and a fundamental basis for subdividing the species [43]. It encodes a two-component signal transduction system that leads to downregulation of surface proteins and upregulation of secreted proteins during in vitro growth. A role for *agr* in virulence has been demonstrated by the attenuated virulence of *agr* mutants in different animal infection models [43].

## Conclusions

The present investigation is the first report of the phenotypic and genotypic analysis of antibiotic resistance in the *S. epidermidis* strains isolated from human hospital infectious samples in Iran. The total prevalence of *S. epidermidis* strains in hospital infectious samples was 46%. Considerable prevalence of resistance against penicillin, tetracycline, erythromycin, cefazolin, and trimethoprim-sulfamethoxazole and high distribution of *aacA-D*, *tetK*, *mecA*, and *tetM* antibiotic resistance genes may pose a potential public health threat. Additionally, *clfA*, *agrIII*, and *etB* were the most commonly detected virulence factors. A high prevalence of multi-drug resistant *S. epidermidis* in the human clinical infectious samples is another important finding of the present study. Moreover, the presence of antibiotic resistance genes and also virulence factors in some *S. epidermidis* strains should be considered as a serious health hazard. Further researches are required to understand additional epidemiological aspects such as the exact relations between antibiotic resistance genes and virulence factors of the *S. epidermidis* strains in hospital infectious samples.

## Methods

### Samples

From February to July 2018, a total of 100 various types of hospital infectious samples were randomly collected from several private hospitals of the Ahvaz city, Iran. Hospital infectious samples were defined as those which were collected from hospitalized patients with severe infections such as UTIs, WIs, and RIs. Furthermore, samples were taken from the site of infection. Samples were immediately transferred to the Clinical Microbiology Research Center of the Islamic Azad University of Shahrekord in a cooler with ice packs.

### Bacterial isolation

*S. epidermidis* was identified by conventional bacteriological tests. The sample was enriched in a tryptic soy broth, and grown on mannitol salt agar, and then catalase, tube coagulase and urease tests, and carbohydrate fermentation were performed. *S. epidermidis* is catalase-positive, coagulase-negative, urease-positive, unable to ferment D-mannitol and D-trehalose, and able to ferment D-mannose and D-maltose [44, 45].

### Antibiotic resistance pattern

Patterns of antimicrobial resistance of the *S. epidermidis* strains were studied using the Kirby-Bauer method. A simple disk diffusion technique on the Mueller-Hinton agar (Merck, Germany) medium was used for this purpose. susceptibility of *S. epidermidis* isolates was tested against several types of antibiotic agents including penicillin (10 μg/disk), cefazolin (30 μg/disk), clindamycin (2 μg/disk), mupirocin (30 μg/disk), azithromycin (15 μg/disk), erythromycin (15 μg/disk), tetracycline (30 μg/disk), ciprofloxacin (5 μg/disk), trimethoprim-sulfamethoxazole (25 μg/disk), nitrofurantoin (300 μg/disk), and rifampin (5 μg/disk) (Oxoid, UK). The instructions of the Clinical and Laboratory Standards Institute were used for this purpose [46]. The plates containing the disks were allowed to stand for at least 30 min before incubated at 37 °C for 24 h. The diameter of the zone of inhibition produced by each antibiotic disc was measured and interpreted using the CLSI zone diameter interpretative standards [46]. *S. epidermidis* ATCC 12228 was used as a quality control organism in antimicrobial susceptibility determination.

### DNA extraction and amplification of virulence factors and antibiotic resistance genes

*S. epidermidis* isolates were sub-cultured on TSB media (Merck, Germany) and further incubated for 48 h at 37 °C. Genomic DNA was extracted from bacterial colonies using the DNA extraction kit (Fermentas, Germany) according to the manufacturer’s instruction. Table 1 represents the list of primers and PCR conditions used for the amplification of virulence factors and antibiotic resistance genes [47]. A programmable DNA thermo-cycler (Eppendorf Mastercycler 5330, Eppendorf-Nethel-Hinz GmbH, Hamburg, Germany) was used in all PCR reactions.

### Statistical analysis

Statistical analysis was done using the SPSS 21.0 statistical software (SPSS Inc., Chicago, IL, USA). Chi-square test and Fisher’s exact two-tailed test were used to assess any significant relationship between the prevalence of *S. epidermidis* strains, virulence factors, and their antibiotic resistance properties. *P* value < 0.05 was considered as statistical significant level.

## Data Availability

All data analyzed during this study are included in this published article.

## Abbreviation

CLSI: Clinical and Laboratory Standards Institute

## References

[1] Ranjbar R, Dehkordi FS, Shahreza MH, Rahimi E (2018). Prevalence, identification of virulence factors, O-serogroups and antibiotic resistance properties of Shiga-toxin producing Escherichia coli strains isolated from raw milk and traditional dairy products. Antimicrob Resist Infect Control..

[2] Ranjbar R, Farsani FY, Dehkordi FS (2018). Phenotypic analysis of antibiotic resistance and genotypic study of the vacA, cagA, iceA, oipA and babA genotypes of the Helicobacter pylori strains isolated from raw milk. Antimicrob Resist Infect Control.

[3] Dehkordi FS, Gandomi H, Basti AA, Misaghi A, Rahimi E (2017). Phenotypic and genotypic characterization of antibiotic resistance of methicillin-resistant Staphylococcus aureus isolated from hospital food. Antimicrobial Resistance & Infection Control.

[4] Ranjbar R, Masoudimanesh M, Dehkordi FS, Jonaidi-Jafari N, Rahimi E (2017). Shiga (Vero)-toxin producing Escherichia coli isolated from the hospital foods; virulence factors, o-serogroups and antimicrobial resistance properties. Antimicrob Resist Infect Control.

[5] Hemmatinezhad B, Khamesipour F, Mohammadi M, Safarpoor Dehkordi F, Mashak Z (2015). Microbiological investigation of O-serogroups, virulence factors and antimicrobial resistance properties of Shiga toxin-producing Escherichia coli isolated from ostrich, turkey and quail meats. J Food Saf.

[6] Momtaz H, Safarpoor Dehkordi F, Taktaz T, Rezvani A, Yarali S (2012). Shiga toxin-producing Escherichia coli isolated from bovine mastitic milk: serogroups, virulence factors, and antibiotic resistance properties. Sci World J.

[7] Atapoor S, Dehkordi FS, Rahimi E (2014). Detection of Helicobacter pylori in various types of vegetables and salads. Jundishapur J Microbiol.

[8] Dehkordi AH, Khaji L, Shahreza MS, Mashak Z, Dehkordi FS, Safaee Y, Hosseinzadeh A, Alavi I, Ghasemi E, Rabiei-Faradonbeh M (2017). One-year prevalence of antimicrobial susceptibility pattern of methicillin-resistant Staphylococcus aureus recovered from raw meat. Trop Biomed.

[9] Madahi H, Rostami F, Rahimi E, Dehkordi FS (2014). Prevalence of enterotoxigenic Staphylococcus aureus isolated from chicken nugget in Iran. Jundishapur J Microbiol.

[10] Momtaz H, Dehkordi FS, Rahimi E, Asgarifar A, Momeni M (2013). Virulence genes and antimicrobial resistance profiles of Staphylococcus aureus isolated from chicken meat in Isfahan province. Iran J Appl Poult Res.

[11] Namvar AE, Bastarahang S, Abbasi N, Ghehi GS, Farhadbakhtiarian S, Arezi P, Hosseini M, Baravati SZ, Jokar Z, Chermahin SG (2014). Clinical characteristics of Staphylococcus epidermidis: a systematic review. GMS Hyg Infect Control.

[12] Oliveira WF, Silva PM, Silva RC, Silva GM, Machado G, Coelho LC, Correia MT (2017). Staphylococcus aureus and Staphylococcus epidermidis infections on implants. J Hosp Infect.

[13] Chu VH, Miro JM, Hoen B, Cabell CH, Pappas PA, Jones P, Stryjewski ME, Anguera I, Braun S, Munoz P, Commerford P (2009). Coagulase-negative staphylococcal prosthetic valve endocarditis—a contemporary update based on the International Collaboration on Endocarditis: prospective cohort study. Heart.

[14] Schaefler S (1971). Staphylococcus epidermidis BV: antibiotic resistance patterns, physiological characteristics, and bacteriophage susceptibility. Appl Microbiol.

[15] Cabrera-Contreras R, Morelos-Ramírez R, Galicia-Camacho AN, Meléndez-Herrada E (2013). Antibiotic resistance and biofilm production in Staphylococcus epidermidis strains, isolated from a tertiary care hospital in Mexico City. ISRN Microbiol.

[16] Klevens RM, Morrison MA, Nadle J, Petit S, Gershman K, Ray S, Harrison LH, Lynfield R, Dumyati G, Townes JM, Craig AS (2007). Invasive methicillin-resistant Staphylococcus aureus infections in the United States. Jama.

[17] Duran N, Ozer B, Duran GG, Onlen Y, Demir C (2012). Antibiotic resistance genes & susceptibility patterns in staphylococci. Indian J Med Res.

[18] Mohaghegh MA, Ghazvini K, Jafari R, Alikhani MY, Safari M, Garamjan A, Ali G, Falahi J, Bordbar D (2015). Retrospective study on the prevalence and antibiotic resistance pattern of staphylococcus aureus and staphylococcus epidermidis among patients suspicious of bacteremia during 2006-2011. Int J Enteric Pathog.

[19] Eladli Mohammed G., Alharbi Naiyf S., Khaled Jamal M., Kadaikunnan Shine, Alobaidi Ahmed S., Alyahya Sami A. (2019). Antibiotic-resistant Staphylococcus epidermidis isolated from patients and healthy students comparing with antibiotic-resistant bacteria isolated from pasteurized milk. Saudi Journal of Biological Sciences.

[20] Ma XX, Wang EH, Liu Y, Luo EJ (2011). Antibiotic susceptibility of coagulase-negative staphylococci (CoNS): emergence of teicoplanin-non-susceptible CoNS strains with inducible resistance to vancomycin. J Med Microbiol.

[21] Cabrera-Contreras R, Morelos-Ramírez R, Galicia-Camacho AN, Meléndez-Herrada E. Antibiotic susceptibility and biofilm production and correlation to methicillin resistant genotype of *Staphylococcus epidermidis* strains from Mexican hospital. In Proceedings of the 110th General Meeting of the American Society for Microbiology (ASM'10). 2010; 23-27.

[22] Delgado S, Arroyo R, Jiménez E, Marín ML, del Campo R, Fernández L, Rodríguez JM (2009). Staphylococcus epidermidis strains isolated from breast milk of women suffering infectious mastitis: potential virulence traits and resistance to antibiotics. BMC Microbiol.

[23] Chaleshtori S, Karimi A (2010). Antibiotic resistance pattern of staphylococcus strains isolated from orange and apple juices in Shahre-kord, Iran. Pakistan J Med Sci.

[24] Raad I, Alrahwan A, Rolston K (1998). Staphylococcus epidermidis: emerging resistance and need for alternative agents. Rev Infect Dis.

[25] Cherifi S, Byl B, Deplano A, Nagant C, Nonhoff C, Denis O, Hallin M (2014). Genetic characteristics and antimicrobial resistance of Staphylococcus epidermidis isolates from patients with catheter-related bloodstream infections and from colonized healthcare workers in a Belgian hospital. Ann Clin Microbiol Antimicrob.

[26] McManus BA, Coleman DC, Deasy EC, Brennan GI, O’Connell B, Monecke S, Ehricht R, Leggett B, Leonard N, Shore AC (2015). Comparative genotypes, staphylococcal cassette chromosome mec (SCCmec) genes and antimicrobial resistance amongst Staphylococcus epidermidis and Staphylococcus haemolyticus isolates from infections in humans and companion animals. PLoS One.

[27] Zelazny AM, Ferraro MJ, Glennen A, Hindler JF, Mann LM, Munro S, Murray PR, Reller LB, Tenover FC, Jorgensen JH (2005). Selection of strains for quality assessment of the disk induction method for detection of inducible clindamycin resistance in staphylococci: a CLSI collaborative study. J Clin Microbiol.

[28] Eksi F (2017). Investigation of antibiotic resistance genes and panton-valentine leucocidin in Staphylococcus aureus strains isolated from various clinical samples. Acta Medica Mediterranea.

[29] Adwan G, Adwan K, Jarrar N, Amleh A (2014). Molecular detection of nine antibiotic resistance genes in methicillin resistant Staphylococcus aureus isolates. Roman Arch Microbiol Immunol.

[30] Johler S, Layer F, Stephan R (2011). Comparison of virulence and antibiotic resistance genes of food poisoning outbreak isolates of Staphylococcus aureus with isolates obtained from bovine mastitis milk and pig carcasses. J Food Prot.

[31] Eftekhar F, Rezaee R, Azad M, Azimi H, Goudarzi H, Goudarzi M (2017). Distribution of adhesion and toxin genes in staphylococcus aureus strains recovered from hospitalized patients admitted to the ICU. Arch Pediatr Infect Dis.

[32] Alfatemi SM, Motamedifar M, Hadi N, Saraie HS (2014). Analysis of virulence genes among methicillin resistant Staphylococcus aureus (MRSA) strains. Jundishapur J Microbiol.

[33] Nowrouzian FL, Dauwalder O, Meugnier H, Bes M, Etienne J, Vandenesch F, Lindberg E, Hesselmar B, Saalman R, Strannegård IL, Åberg N (2011). Adhesin and superantigen genes and the capacity of Staphylococcus aureus to colonize the infantile gut. J Infect Dis.

[34] Lim KT, Hanifah YA, Mohd Yusoff MY, Thong KL (2012). Investigation of toxin genes among methicillin resistant Staphylococcus aureus strains isolated from a tertiary hospital in Malaysia. Trop Biomed.

[35] Jiménez JN, Ocampo AM, Vanegas JM, Rodríguez EA, Garcés CG, Patiño LA, Ospina S, Correa MM (2011). Characterisation of virulence genes in methicillin susceptible and resistant Staphylococcus aureus isolates from a paediatric population in a university hospital of Medellin, Colombia. Mem Inst Oswaldo Cruz.

[36] Sila J, Sauer P, Kolar M (2009). Comparison of the prevalence of genes coding for enterotoxins, exfoliatins, panton-valentine leukocidin and tsst-1 between methicillin-resistant and methicillin-susceptible isolates of Staphylococcus aureus at the university hospital in Olomouc. Biomed Pap Med Fac Univ Palacky Olomouc Czech Repub.

[37] Demir C, ASLANTAŞ Ö, Duran N, Ocak S, ÖZER B (2011). Investigation of toxin genes in Staphylococcus aureus strains isolated in Mustafa Kemal University Hospital. Turk J Med Sci.

[38] Ghasemian A, Najar Peerayeh S, Bakhshi B, Mirzaee M (2015). Several virulence factors of multidrug-resistant Staphylococcus aureus isolates from hospitalized patients in Tehran. Int J Enteric Pathog.

[39] Almeida LM, de Almeida MZ, Mendonça CL, Mamizuka EM (2013). Comparative analysis of agr groups and virulence genes among subclinical and clinical mastitis Staphylococcus aureus isolates from sheep flocks of the northeast of Brazil. Braz J Microbiol.

[40] Zhang Y, Xu D, Shi L, Cai R, Li C, Yan H (2018). Association between agr type, virulence factors, biofilm formation and antibiotic resistance of Staphylococcus aureus isolates from pork production. Front Microbiol.

[41] Cheung GY, Wang R, Khan BA, Sturdevant DE, Otto M (2011). Role of the accessory gene regulator agr in community-associated methicillin-resistant Staphylococcus aureus pathogenesis. Infect Immun.

[42] Nasirian S, Saadatmand S, Goudarzi H, Goudarzi M, Azimi H (2018). Molecular investigation of methicillin-resistant Staphylococcus aureus strains recovered from the intensive care unit (ICU) based on toxin, adhesion genes and agr locus type analysis. Arch Clin Infect Dis.

[43] Gomes-Fernandes M, Laabei M, Pagan N, Hidalgo J, Molinos S, Hernandez RV, Domínguez-Villanueva D, Jenkins AT, Lacoma A, Prat C (2017). Accessory gene regulator (Agr) functionality in Staphylococcus aureus derived from lower respiratory tract infections. PLoS One.

[44] Tille PM. Bailey & Scott's Diagnostic Microbiology. 14th edition. London: Mosby; 2018. pp. 254-3.

[45] Topley WWC, Wilson GS (2005). Topley & Wilson Microbiology and Microbial Infection.

[46] CLSI (2017). Performance standards for antimicrobial susceptibility testing; twenty-seven informational supplement. CLSI document M100-S26.

[47] Momtaz H, Hafezi L (2014). Meticillin-resistant Staphylococcus aureus isolated from Iranian hospitals: virulence factors and antibiotic resistance properties. Bosnian J Basic Med Sci.

